# Making the journey with me: a qualitative study of experiences of a bespoke mental health smoking cessation intervention for service users with serious mental illness

**DOI:** 10.1186/s12888-016-0901-y

**Published:** 2016-06-08

**Authors:** Sarah Knowles, Claire Planner, Tim Bradshaw, Emily Peckham, Mei-See Man, Simon Gilbody

**Affiliations:** NIHR School for Primary Care Research and Manchester Academic Health Science Centre, University of Manchester, Manchester, M13 9PL UK; School of Nursing, Midwifery and Social Work, University of Manchester, Manchester, M13 9PL UK; Department of Health Sciences, University of York, York, YO10 5DD UK; School of Social and Community Medicine, University of Bristol, Bristol, BS8 2PS UK

## Abstract

**Background:**

Smoking is one of the major modifiable risk factors contributing to early mortality for people with serious mental illness. However, only a minority of service users access smoking cessation interventions and there are concerns about the appropriateness of generic stop-smoking services for this group. The SCIMITAR (Smoking Cessation Intervention for Severe Mental Ill-Health Trial) feasibility study explored the effectiveness of a bespoke smoking cessation intervention delivered by mental health workers. This paper reports on the nested qualitative study within the trial.

**Methods:**

Qualitative semi-structured interviews were conducted with 13 service users receiving the intervention and 3 of the MHSCPs (mental health smoking cessation practitioners) delivering the intervention. Topic guides explored the perceived acceptability of the intervention particularly in contrast to generic stop-smoking services, and perceptions of the implementation of the intervention in practice. Transcripts were analysed using the Constant Comparative Method.

**Results:**

Generic services were reported to be inappropriate for this group, due to concerns over stigma and a lack of support from health professionals. The bespoke intervention was perceived positively, with both practitioners and service users emphasising the benefits of flexibility and personalisation in delivery. The mental health background of the practitioners was considered valuable not only due to their increased understanding of the service users’ illness but also due to the more collaborative relationship style they employed. Challenges involved delays in liaising with general practitioners and patient struggles with organisation and motivation, however the MHSCP was considered to be well placed to address these problems.

**Conclusion:**

The bespoke smoking cessation intervention was acceptable to service users and the both service users and practitioners reported the value of a protected mental health worker role for delivering smoking cessation to this group. The results have wider implications for understanding how to achieve integrated and personalised care for this high-risk population and further underscore the need for sensitised smoking cessation support for people with serious mental illness.

**Trial registration:**

Current Controlled Trials ISRCTN79497236. Registered 3^rd^ July 2009.

## Background

Service users with serious mental illness (SMI) have higher rates of morbidity and premature mortality compared to the general population, suffering the loss of an estimated 13–20 years of life compared to those without SMI [1]. These excess mortality rates are largely due to modifiable risk factors such as smoking, but there are recognised barriers at the individual, health care professional and systems level to addressing these problems in this population [2–4]. The National Audit of Schizophrenia in 2012 reported that the most serious deficits in care for people with psychosis were in the monitoring and management of their physical health problems [5]. There is, therefore, an urgent need to identify methods for improving the assessment and management of physical health problems in service users with SMI and to provide comprehensive preventative focussed services.

Smoking is recognised as one of the major modifiable contributors to early mortality for this population [6]. Tobacco related conditions are estimated to comprise approximately 53 % of all deaths of people with schizophrenia and 48 % of people with bipolar disorder [7]. People with mental health problems consume 42 % of the tobacco used in England and within this group people with psychosis show one of the highest smoking rates, with smoking suggested as a core factor responsible for their health inequalities [8]. There is a higher prevalence of smoking in people with psychosis (up to 80 % in people with schizophrenia [9]) compared to the general population, and people with psychosis are more likely to be classed as heavy smokers [10], therefore representing an especially high risk group to be targeted by smoking cessation programmes. Consequently, addressing smoking in this population is recognised as of major clinical importance. In the UK, the Royal College of Psychiatrist’s 2013 report “Whole Person Care: Achieving parity between mental and physical health” argued that parity could be achieved for people by tackling the premature mortality rates of people with mental illness, and specifically advised commissioners to ensure that a major focus of their smoking cessation services was on smokers with mental health problems [11].

However, despite being as motivated to stop smoking as the general population, only a minority of those with mental illness receive smoking cessation interventions [12]. This may be due to commonly held misconceptions that people with mental illness are unwilling or unable to quit, with reports that services have ‘low aspirations’ in relation to their smoking status [11]. Opportunities to engage service users with smoking cessation programmes may therefore be missed, further exacerbating disparities in care. Beyond the negative impact on health outcomes, failure to address smoking behaviour is also discriminatory and neglects the desire and the right of service users with SMI to enter smoking cessation programmes [10]. As well as barriers to accessing support, there are also concerns about the suitability of traditional smoking cessation programmes for this population [13, 14], with the fragmentation of mental and physical health services further impeding attempts to provide holistic physical and mental health care.

The SCIMITAR feasibility trial aimed to address these barriers through the provision of a ‘bespoke smoking cessation’ (BSC) service, where “bespoke” refers to the specific tailoring of the intervention to people with severe mental illness. The BSC was an individually tailored service delivered by a skilled mental health practitioner that aimed to work in conjunction with the participant and the participant’s family doctor or mental health specialist. The intervention met NICE guidelines for smoking cessation services at the time of the trial and was delivered according to the Manual of Smoking Cessation (guide for Counsellors and Practitioners [15]) which forms the basis of client-centred smoking cessation interventions in the NHS via the National Centre for Smoking Cessation Training (NCSCT, http://www.ncsct.co.uk). In the SCIMITAR trial the intervention was further adapted to meet the particular needs of his population. Standard NHS smoking cessation includes elements of personalisation, with tailoring of content to the individual recommended in best practice guidelines [16], but the BSC aimed to specifically tailor content and delivery for people with serious mental illness. As well as delivery by a mental health professional, this included, for example, providing additional face-to-face support following relapse, recognising the motivations for smoking in the context of their mental illness, a focus on home visits and collaboration with other professionals involved in the service user’s care.) Full details of the intervention are reported in both the main trial paper and an accompanying paper with case study examples of the MHSCPs work [17, 18]. The trial found that smoking cessation was highest amongst individuals receiving the BSC service and participants engaged well with the service, in contrast to those in the usual care group, none of whom accessed NHS smoking cessation services during the trial. It should be noted however that the trial did not demonstrate a significant difference between the intervention and control arms. However, the trial was a feasibility study, intended to establish the feasibility of recruiting participants and delivering the intervention, and was not intended to demonstrate effectiveness. A fully powered trial is underway which will establish this (funded by NIHR Health Technology Assessment, reference HTA 11/136/52.)

The aim of the present study was to qualitatively explore the experiences of service users who received the BSC intervention, particularly in comparison to their experience of standard stop-smoking services and in light of their mental health difficulties. We also interviewed the Mental Health Smoking Cessation Practitioners (MHSCPs) who delivered the intervention at each site, to explore their attitudes toward implementing their training in the delivery of the intervention and to intervention content.

## Methods

Sampling was purposive, with invitations sent to participants in the intervention arm of the trial in order to explore experience of receiving the intervention. We furthermore aimed to purposively sample both service users who had completed treatment with their MHSCP and those who had withdrawn from treatment, Service users who had withdrawn were specifically invited in order to ensure that factors impacting on disengagement with the BSC were captured and because we anticipated that disengagement would be an indicator of low acceptability, and this would ensure we captured both positive and negative views.. 15 participants responded to the initial invitation and expressed an interest in participating and 13 were interviewed (the remaining 2 participants were not interviewed due to difficulties arranging the interviews within the time frame of the study.) Interviews took place between August 2012 and January 2013. Ethics approval was sought and granted on Oct 29, 2010, by Leeds (East) Research Ethics Committee (10/H1306/72). Participants provided written consent prior to interviews taking place. After completion of the interview, as a token of thanks for their time, the participant was offered a £10 gift voucher. Participants were not notified of this voucher until after the interview had been conducted so as not to be coercive or cause undue influence over the participant’s responses. Interviews were digitally recorded and transcribed verbatim with permission of the participants.

The topic guides were developed by the first three authors and explored:

- Prior experience of smoking cessation, including support received from other primary care or mental health professionals.
- Acceptability of the intervention and satisfaction with the bespoke smoking cessation service (particularly in comparison to previous smoking cessation interventions received).
- Service users’ engagement with the intervention; with specific reference to barriers and facilitators to compliance with smoking cessation interventions.
- Implementation in routine care, including perceptions of who is best to deliver the BSC, any anticipated barriers to implementation.

### Sample

13 service users were recruited from across the 3 recruitment sites (5 from Manchester, 6 from York and 2 from Hull). 3 MHSCPs, one from each site, were interviewed.^1^ All 3 MHSCPs were female, had experience working in Community Mental Health settings and none had previous experience or training of smoking cessation. Of the 13 service users, 2 were female and the average age was 50 (range 32–68). Only 2 participants were formally considered to have disengaged from the intervention (had deliberately expressed a wish to withdraw or to discontinue with the service). However, the interviews with both service users and MHSCPs indicated that sustaining engagement was problematic for all participants and that withdrawal was not directly related to acceptability in the way we had anticipated. All participants expressed struggles with engagement (The issue of engagement is reported in Theme 3.) rather than a subset of patients withdrawing deliberately due to negative perceptions of the intervention. Consequently we finalised data collection once we believed data saturation had been reached rather than attempting to further sample according to the original framework.

Participants in the qualitative sample had a similar profile of smoking history and quit attempts to the trial population overall [17] – the sample participants had smoked for an average of 32 years and had tried to quit 5 times, with full trial sample having an average age of 27.1 and also an average 5 quit attempts. All of the participants in the study were White British. Regarding diagnosis, 5 of the service users had Bipolar disorder, 6 had Schizophrenia (3 reported Paranoid Schizophrenia) and 2 had Depression with Psychosis.

### Analysis

Transcripts were read independently by two authors (SK and CP) and analysed using the Constant Comparison method [19]. Constant comparison aims to inductively identify themes through categorising and coding data and exploring connections between them, repeating the cycle across the data set until theoretical saturation is achieved. Emergent themes were discussed and verified with a third author (TB). Analysis was finalised prior to the completion of the quantitative analysis and was therefore blind to study outcome.

The MHSCPs transcripts were initially analysed independently from the patient transcripts, but the analysis was combined when preliminary readings suggested consensus in core themes across the two data sets. We also observed that novel insights could be synthesised across the two samples to provide a holistic picture of the intervention, with complementary perspectives and insights on acceptability of the interventions and challenges encountered. Similarly, the transcripts of participants that completed the intervention and those that disengaged were analysed together and synthesised to represent challenges to engagement with smoking cessation, rather than exploring differences between subsamples. Although only 2 of the participants were formally considered to have ‘disengaged’ (having been discharged due to a lack of contact with the MH-SCP), both service users reported positive experiences with the intervention itself, suggesting other circumstances may have contributed to their disengagement, and even service users considered to have engaged with treatment reported difficulties maintaining motivation and planning future sessions. It was therefore felt that these data were best captured through over-arching themes rather than treated as independent.

## Results

We identified three main themes, reflecting the problems that service users with SMI encountered in terms of smoking cessation in routine care, the perceived benefits of the bespoke intervention for this population, and finally barriers to the intervention in practice.

### Theme 1: generic stop smoking services experienced as unsuitable, and a lack of support from health professionals for smoking cessation for service users with SMI

Participants reported a lack of support for smoking cessation in current primary care and mental health services, both implicitly through a lack of support for cessation or through services appearing not to prioritise smoking cessation for this group, and explicitly in the form of professionals advising against quit attempts.

“When I go for an appointment and if they’re catching up on their records, and it comes to smoking and they say how many are you smoking, do you still smoke. I say yeah, and they say, you know you should really start trying to give up now, don’t you? You know, just things like that. There’s nothing based upon a reason to encourage me not to”. M1024

“Doctors are always recommending me to give up smoking. Yes. I can’t really remember what they said. They just say, ‘Do you smoke?’ And I say yeah, and they said, ‘Give up’. And the nurses, they say we can help you but that’s all they say. It’s sort of…they can give out patches but the only really advice they give is, one time they said, ‘Now really try this time, [patient name]. Really try’. And I was like alright. They just signed the prescription and I take it away and try, so that was that”. M1037

In other cases, health professionals actively discouraged smoking cessation attempts, either due to concerns about the impact on service users’ mental health or due to a perception that prescription only stop-smoking medications are unsuitable for people with SMI:

“I’ve actually had a doctor turn round and say, after quite an episode which was quite a lengthy episode, and I talked about giving up, he said, oh no, you don’t want to be giving up at the moment. So it was kind of like a medical permission to carry on smoking… The doctor might say, as he said, terrible thing smoking. But never actually say, you should give up, and I’ll refer you. I’ve had to ask for that. The last thing you want to think about is giving up, that sort of comment comes across”. Y1085

“[The practice nurse] just simply said, “We’re not putting you on the Champix”, and the other one as well, “Not putting you on them”. And that was it. I was out the door, gone”. M1100

The MH-SCPs themselves acknowledged a lack of support for smoking cessation in people with SMI delivered through existing services:

“Even though I knew it was an issue, it’s not something that had really been at the forefront of my mind, and thought that we necessarily should be really targeting and addressing. So that did make me rethink about my own practice, and about how we do approach that with people… when I did the training, it did make me think more that actually we don’t do enough, and there is ways in with people. And aware that I’ve been guilty in the past, especially when working in acute in-patient units, thinking that maybe this isn’t the best time for people to give up smoking, and giving those messages out”. MHSCP1

### Theme 2: the benefits of the MH-SCP role and the bespoke intervention for service users with SMI

The perceived benefits of the MH-SCP included both the bespoke nature of the intervention, with the practitioner able to personalise the treatment to the individual and their circumstances, and the sensitisation of the intervention to the particular mental health needs of the individual.

#### Providing a bespoke smoking cessation intervention

The bespoke nature of the intervention enabled personalisation to service users, both in terms of their individual needs (such as whether they preferred to cut down or quit, preferring visits at home or elsewhere), their condition (their mental health diagnosis, symptoms and medications) and their specific health care context (in terms of working with the existing network of care co-ordinators, GPs and nurse prescribers and psychiatrists).

“You work flexibly, they get someone that’s got some understanding of their mental health issues, someone who can work with, you know, have the time to work with the other network of people that are involved with them as well”. MHSCP 3

“It was individual to the person really, flexible to their needs, like seeing them when they wanted within reason and then not putting too much pressure on them…just tailored to the person see what works for each person… It was interesting how each person was completely different what they wanted to do and what they wanted from me and how motivated they were and everything…you can’t just say ‘I’ve got to read this script’”. MHSCP2

“there’s a mental health history, sort of checking out who their key workers are, where they feel they are with their mental health at that time, and the psychiatric medication that they’re taking…stopping smoking can have an impact on certain medications so it was needing to know that, checking out that they were okay for me to liaise with any mental health workers, GPs et cetera. And it was just really getting a feel for where they were at”. MHSCP 1

#### Providing smoking cessation sensitive to the mental health needs of people with SMI

In regard to sensitisation, service users appreciated the greater mental health knowledge and awareness of the MH-SCPs, particularly in contrast to routine services where service users reported anxiety about stigma attached to their condition:

“It wasn’t just a stop smoking clinic for Tom, Dick and Harry, she understood the mental health side, which is obviously a big concern… Because I wouldn’t go to a normal - because I’m frightened…Well [the MHSCP] knows what I’ve got. Whereas if you go to a normal stop smoking thing and they know you’ve got mental health problems then it’s stigma isn’t it?… you’ve got to trust the person who you’re talking to and be comfortable with them, especially on mental health issues”. H1098

The MH-SCPs also reported that service users they saw had struggled with generic services being inappropriate and with concerns about stigma:

“I did have one chap that came…and he’d been to normal standard NHS services, to a group, and he had a diagnosis of bipolar, and … she’d given them all a prescription request sheet for Champix. He went to see his GP and his GP said, ‘I’m not giving you Champix, you’ve got bipolar’. So he came back next week, and he was the only one in the room that hadn’t been given the Champix. And he said he felt really awkward. ‘How do I explain why I couldn’t have the Champix?’ He said, ‘I didn’t want to tell them it’s because I had a mental health problem’”. MHSCP 1

The appreciation of the mental health background of the MH-SCPs was not purely knowledge based however – service users also emphasised the benefits of the more collaborative or compassionate style of working that they attributed to the mental health background of the practitioners, again in contrast to their experiences with generic stop-smoking services in Primary Care:

“The nurses, they don’t give you much time to talk about it really. They just sort of pack you off with some boxes of patches. [The MHSCP] listens to your mental health problems as well, what you’re thinking…she helped me to… feel at ease about not being so hard on myself again if I’m suffering from illness…she gave me a lot of peace of mind”. M1037

“I found that the relationship I had with [the MHSCP], was such that she was supportive without pushing. And it’s very much the case that she was there to help, for advice, rather than to ram anything down my throat…It becomes more of a therapeutic relationship, rather than the nurses making me, or the nurses leading me, whereas in a therapeutic relationship, it’s the nurses walking along beside me, making the journey with me rather than pushing me”. Y1053

The MHSCPs themselves also reported that their mental health background enabled a more holistic approach to smoking cessation:

“I think you can train anybody to deliver smoking cessation, but it doesn’t work if you don’t understand the issues of people with serious mental illness…you can’t deliver it cold, you can’t just work on smoking with somebody with a serious mental illness, if they’re complex, they’ve got lots of other things going on, you have to take them as a whole”. MHSCP 3

“It’s about engagement as well, that therapeutic alliance, it’s all that sort of stuff. If somebody’s got that with somebody and they have the smoking cessation skills as well, then I think they’re best placed to support that person”. MHSCP 1

### Theme 3: reported challenges and barriers

The main reported challenges were at the service level (concerning interactions with Primary Care) and at the patient level (concerning difficulties sustaining engagement).

#### Service level barriers – integration with primary care

MH-SCPs reported that smoking cessation work was best delivered by someone with protected time and space for the role, which would be difficult to achieve in current routine care:

“You could put this work into the mainstream, you know, into CPNs [Community Psychiatric Nurses] work, but I don’t know that everybody would do it, that’s the thing, and how much time and attention they would give, because you need to be quite focused”. MHSCP 3

“Whether if they said to people in CMHTs just get somebody who does a specific smoking cessation speciality I don’t know if it would work because say at [Community Residential Unit] they had a smoking cessation worker there who I met and I’m like ‘Well why am I here like?’ And it’s because her role just was eclipsed and she was just doing the general support work. So you’d have to have a specific… you’d have to be quite regimented in doing your work”. MHSCP 2

The practitioners also reported some barriers to working in Primary Care, specifically around effectively liaising with GPs, which was also reported by service users and which could cause delays to treatment:

“If the GP wouldn’t prescribe… then you’re chasing it up and then when the client goes it’s not there and they get annoyed that they’ve wasted a visit to the doctors. Some GP surgeries refused to do it on my recommendation and had to see the client. So then the client had to make an appointment with the GP which just didn’t happen. So then I’d say well I’ll give you a letter to take with the doc… and then they lose the letter”. MHSCP2

“I would have said, if anything, my own doctors let [the MHSCP] down because she would put things in to request for things that I needed, but they weren’t coming through quick enough… I think we used to sometimes do texts, can I just check, have you spoken to my doctor? And she’d say, I’ve written the letter. And I’d go across and try and pick up my prescription, and it just wouldn’t be ready”. H1066

It was nevertheless emphasised that the MH-SCP role was best placed within primary care, and that mental health workers were uniquely placed to bring together primary and secondary services for this group:

“A lot of the people with serious mental illness are now seen in general practice and nowhere else…so people are handed back to general practice, to benefit the most people, there’d have to be something done in primary care”. MHSCP3

“Well they’ve [mental health workers] got the skills, they’ll already be working with the client group, they’ll have the contacts, they’ll have the links…I think it will fit together better. Because I think this should be integral rather than seen as a separate service”. MHSCP 1

#### Service-user level barriers – motivation and disorganisation

Both service users and practitioners acknowledged a core problem around maintaining engagement and motivation. Although all the service users who joined the trial had expressed a desire to cut down or quit smoking, sustaining their engagement, particularly during or after condition relapses could be problematic and service users themselves reported the need for support to be accessible when the ‘window of opportunity’ was open:

“It [starting the intervention] was over Christmas, and before Christmas I really, really wanted to quit, and I was ready to quit. But when I saw [the MHSCP], I don’t think I was ready to quit… When things get a bit rough, I start smoking. And that really [happened] actually about a couple of month before I started seeing [the MHSCP]. If I’d have started seeing her in the first place, it would have been a different tale. I would have quit, and I know I would. Timing, timing. Getting the timing right”. Y1084

“I’d started to go through a little bit of an up and downer, I felt as though I took it serious and I wanted to do it, and then something would come along and sort of like take my mind off everything… I just lost all, you know, so it weren’t the fault of anybody, other than the mind of me”. H1066

Other service users who struggled with cognitive or memory problems also had difficulties sustaining their smoking cessation:

“It was working and then all of a sudden I’m smoking 20 cigarettes a day, and it's like, to be honest, I can't remember it … a lot of my memories from then are very cloudy. I can't remember in detail things like, you know, like you're asking, why did I start smoking again”. M1024

The MH-SCPs reported that this problem was complicated by the often chaotic lifestyles of people with SMI and their difficulties in organising and adhering to a smoking cessation plan:

“She disengaged and was texting me saying, ‘Oh I’ve not done too well this week so can you come next week?’ And I’d go and she wouldn’t be there… even if I could say only one of my clients attended every appointment [but] none of them did…I think it’s reflective of the patient group really…. they’re just so chaotic, very few of them had diaries and if they did it wasn’t really like a diary it was a notebook that was all upside down… they’d just write on one page that you were coming and then they just put it in a drawer”. MHSCP 2

However this problem was seen as reaffirming the need for the personalised and proactive care that the MHSCP could provide:

“She would lose the prescription, the house was, you know, quite chaotic, she’d lose them, then she’d think she’d run out of them and she’d get muddled with them, so I had to do quite a bit of work around that really, I mean, if I went in her house now, I know exactly where she keeps everything and where she loses everything! I don’t know if that was my role, but it helps!”. MHSCP 3

“Mentally ill people probably do benefit from this service because it's not that they can't think for themselves in the same way as other people, but it's more a case of they can't organise themselves or their thoughts in the same way as other people. So they probably just need that little bit of extra help”. M1024

Participants emphasised that problems with motivation should not be equated with not wanting to give up, and that flexibility allowing them to re-engage would be important:

“Probably [it’s] because at that time, it's too much for them to add to what's going on in their lives … you don't completely abandon them … So maybe if you just keep… keep going back to them. You're saying look, we're here and we'll keep letting you know we're here”. M1024

## Discussion

The study explored the acceptability of a bespoke smoking intervention for people with SMI though interviews with participants receiving the intervention and practitioners trained to deliver it. The data suggest that the intervention has the potential to increase both access to and acceptability of smoking cessation for this high risk group. The findings offer confirmation that generic smoking cessation services are likely to be unsuitable for this group, and that currently there is a lack of support for smoking cessation offered in Primary Care. Participants reported that health professionals could explicitly discourage quit attempts, consistent with concerns about diagnostic and treatment overshadowing for this group [20], which refers to the tendency for services and health professionals to prioritise management of one conditions (in this case SMI) at the expense of others (physical health).

The mental health sensitised intervention offered here was perceived as more appropriate and acceptable for service users. The bespoke nature of the intervention enabled practitioners to tailor the intervention to individuals in terms of both their mental health and medication status and their individual preferences and levels of motivation. It was notable that both participants and practitioners considered the mental health background of the MH-SCPs to be important not only in terms of understanding medication needs or avoiding the anxiety around stigma (both of which reduced the acceptability of generic services), but also for the more collaborative and supportive relationship style that the MHSCPs employed and which was considered essential for working effectively with this patient group. It is well established in the literature on dual diagnosis that additional support and assertive outreach are crucial components of tailoring interventions to this particular population [21]. The study reported here demonstrates that service users perceived the MH-SCPs as particularly suited to building these more supportive relationships with them and that they are equally valued in the context of smoking cessation.

The MHSCPs also emphasised however the importance of a protected space with protected time to focus on smoking cessation outside of routine support work typically provided by mental health workers and felt that the role was best placed within primary care. It is recognised that people with SMI can be disadvantaged by fragmented care and risk ‘falling through the gaps’ [22] and the data demonstrate how MHSCPs, if given a protected space to focus on smoking cessation, were able to liaise between primary and secondary services. Given that one-third of people with SMI are only seen in primary care [23], this finding supports the need for physical health initiatives for people with SMI to be integrated within primary care.

Both service users and practitioners acknowledged that wavering motivation levels and difficulties in organisation were a particular problem for this group. The need to provide the intervention at ‘the right time’ suggests that maintaining open access to the intervention is necessary so that service users can re-engage with treatment when they are ready, but there are clearly questions around whether such a model is sustainable in routine care. It may be more pragmatic for future research to explore whether explicit motivational or organisational support, for example through provision of text based reminders (which has been demonstrated to improve anti-psychotic medication adherence [24]) could be built into the interventions to maintain engagement. Another implication of this finding however, is that problems with motivation and engagement should not be construed as reflecting unwillingness to reduce or quit smoking, but rather reflect the difficulties faced by this group in maintaining abstinence or a reduced rate of smoking over time.

### Limitations

Firstly, the included sample size is small. However, the emergent themes were highly consistent across participant and professional reports which support the robustness of the findings. Secondly, inevitably participants in the study reflect those who wished to engage with smoking cessation services and findings may not generalise to other service users with more complex needs and/or lower levels of motivation. However, the aim of the trial was to provide bespoke smoking cessation to service users who requested treatment, not address motivation to quit for those who may be unwilling, and motivation was revealed to be complex and fluctuating even amongst this group.

Thirdly, while service users in the study were positive about their experiences with the MHSCPs, this may reflect that the practitioners who volunteered for the training were especially motivated or reflect the relatively small number of cases each MHSCP had to manage. The MHSCPs themselves also questioned whether their role was feasible within routine care if protected space could not be maintained. It will be important in larger trials of such interventions to determine if the experiences of the practitioners in the pilot study generalise to larger cohorts of MHSCPs and also to address implementation within everyday workloads within existing care services.

Finally, in terms of generalisability, only 2 of the interviewed participants were female compared to 40 % in the overall trial population, suggesting women were underrepresented in the study. The sample was also exclusively White British, indicating that further work is necessary to explore the acceptability of a bespoke intervention to other ethnic groups.

### Implications

Better implementation of physical health care for people with serious mental illness is a recognised priority internationally, with a need for greater understanding of how to deliver integrated physical health programmes effectively for this population [25]. In the UK, the Mental Health Foundation report “Crossing Boundaries” identified nine areas of good practice which could be targeted to achieve more integrated care, but highlighted that having “*staff who understand the holistic nature of health care and have no professional defensiveness about working closely with colleagues in other disciplines, and with patients and families*” (p7) were key to quality integrated care [26]. The data collected here demonstrates what this may look like in practice for physical health initiatives for service users with psychosis. This would involve staff, trained in delivery of both physical and mental health interventions, who are able to effectively liaise between primary and secondary care services, and who also commit to working flexibly and sensitively with service users with complex needs. The evidence reported here can complement initiatives such as ReThink’s physical health pathway [27] through the identification of professional and service level issues that could potentially hinder the implementation of such initiatives in practice.

## Conclusions

Service users with SMI are often excluded from typical stop-smoking services, either due to such services being inappropriate for them or due to a lack of support to engage with smoking cessation from health professionals. The findings reported here demonstrate that, although service users with SMI can struggle to sustain motivation and engage with treatments, they are willing to engage with smoking cessation practitioners who can understand their mental health problems and who are able to work flexibly and collaboratively with both the patient and with others involved in their care.

## Footnotes

1 Manchester and York each had one MH-SCP for the duration of the study. Hull had two MH-SCPs. The second Hull practitioner was unavailable for interview due to emigration.
